# Clinical outcomes of hospitalised COVID-19 patients at Mthatha Regional Hospital, Eastern Cape, South Africa: A retrospective study

**DOI:** 10.4102/safp.v63i1.5253

**Published:** 2021-06-10

**Authors:** Ramprakash Kaswa, Parimalaranie Yogeswaran, Busisiwe Cawe

**Affiliations:** 1 Department of Family Medicine and Rural Health, Faculty of Health Sciences, Walter Sisulu University, Mthatha, South Africa

**Keywords:** COVID-19, diabetes, hypertension, hospitalised, co-morbidity

## Abstract

**Background:**

Coronavirus disease 2019 (COVID-19) is a public health crisis that threatens the current health system. The sudden expansion in the need for inpatient and intensive care facilities raised concerns about optimal clinical management and resource allocation. Despite the pressing need for evidence to make context-specific decisions on COVID-19 management, evidence from South Africa remained limited. This study aimed to describe the clinical characteristics and outcomes of COVID-19 hospitalised patients.

**Methods:**

A retrospective cross-sectional study design was used to evaluate the clinical outcomes of hospitalised adult patients (≥ 18 years old) with laboratory-confirmed COVID-19 illness at Mthatha Regional Hospital (MRH), Eastern Cape.

**Results:**

Of the 1814 patients tested for COVID-19 between 20 March 2020 and 31 July 2020 at MRH, two-thirds (65.4%) were female. About two-thirds (242) of the 392 patients (21.6%) who tested positive for this disease were hospitalised and one-third (150) were quarantined at home. The mean age of the patients tested for COVID-19 was 42.6 years and there was no difference between males and females. The mean age of hospitalised patients was 55.5 years and the mean age of hospitalised patients who died (61.3 years) was much higher than recovered (49.5 years). Overall, 188 (77.6%) hospitalised patients had clinical comorbidity on admission. Diabetes (36.8%) and hypertension (33.1%) were the most common comorbidities amongst COVID-19 hospitalised patients.

**Conclusion:**

The majority of the patients who were hospitalised for COVID-19 were elderly and had high baseline comorbidities. Advance age and underlying comorbidities (diabetes, hypertension and HIV) were associated with high mortality in hospitalised COVID-19 patients.

## Introduction

Coronavirus disease 2019 (COVID-19) is a public health crisis that threatens the current healthcare system.^1^ It was first identified in Wuhan, Hubei Province, China, in December 2019.^2^ Throughout the world, about 82 million cases were reported, and close to 1.8 million people died from COVID-19 by December 2020.^3^

The virus is primarily transmitted by inhalation or contact with infected droplets. Currently, available data indicate that the majority of people with the disease have mild symptoms, whilst about 20% develop moderate-to-severe disease. About 5% of these may develop severe pneumonia, acute respiratory distress syndrome and multi-organ dysfunction.^4^

In South Africa, the first case of COVID-19 was confirmed on 05 March 2020. Since then about 1 million laboratory-confirmed cases and about 27 000 deaths (as of December 2020) have been reported across the country.^5^ The rapid spread of COVID-19 has heavily taxed the current health system, resulting in a shortage of health resources for COVID-19 management throughout the country. The high burden of the disease quickly exceeded the standard capacity of healthcare systems. The sudden expansion in the need for inpatient and intensive care facilities raised concerns about optimal clinical management and resource allocation.^6^ Despite the pressing need for evidence to make context-specific decisions on COVID-19 management, evidence from South Africa remained limited.

Clinically, COVID-19 presents with flu-like symptoms of fever, sore throat, dry cough, headache, dyspnoea, general malaise and gastrointestinal symptoms such as diarrhoea.^7^ Other clinical features include loss of smell and taste sensations. Severe cases present with signs of pneumonia and acute respiratory distress syndrome.^8^ The case fatality rate of severe acute respiratory syndrome coronavirus 2 (SARS-CoV-2) is lower than that of its two predecessors. However, the highly infectious nature of SARS-CoV-2 is a big challenge amongst critically ill patients.

Being elderly and having comorbidities such as diabetes, hypertension, chronic kidney disease, autoimmune conditions and cancer are associated with a severe form of the disease and high mortality.^9^ The South African population is generally younger but potentially at higher risk because of the high burden of non-communicable diseases (NCDs). In addition, HIV and tuberculosis (TB) epidemic coincided with the COVID-19 pandemic in South Africa.^7^ Although there is evidence to suggest the severity of COVID-19 in patients with NCDs, there is limited data on the effect of HIV, TB and other prevalent chronic medical conditions on the clinical outcome of COVID-19.

The full impact of this new pandemic on the health, social and economic well-being of humankind is still uncertain.^10^ To date, there is no recommended curative treatment, and supportive measures of oxygen therapy and corticosteroids are the only ways available for the control of COVID-19. Several explanations have been hypothesised for this unexpected high care fatality amongst COVID-19 hospitalised patients in South Africa, including a high concomitant HIV epidemic, delayed presentation and a relatively weak health system. However, in South Africa, there are limited data on the prevalence of comorbidity with NCDs (e.g. hypertension, diabetes and obesity) and communicable diseases (e.g. HIV and TB) amongst SARS COVID-19 hospitalised patients, which may influence COVID-19 presentations and outcomes. Furthermore, much remains unknown about these patient’s clinical care, outcomes and the required resources in the South African context. This study aimed to describe the clinical characteristics and outcomes of COVID-19 hospitalised patients. The objectives of the study were to describe the demography, comorbidities and clinical outcomes of COVID-19 at Mthatha Regional Hospital (MRH).

## Methods

### Study design and participants

A retrospective cross-sectional study design was used to evaluate the clinical outcomes of hospitalised adult patients (≥ 18 years old) with laboratory-confirmed COVID-19 illness at MRH, Eastern Cape.

**FIGURE 1:**
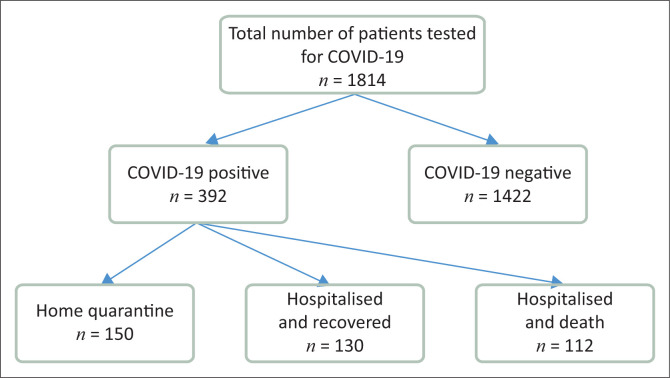
Flow chart of patients tested for COVID-19 at Mthatha Regional Hospital.

The study was carried out from 20 March 2020 to 31 July 2020 and all adult patients who were diagnosed with COVID-19 and hospitalised as inpatients to MRH were included in the study. Figure 1 demonstrates the study participants.

### Study setting

The study was conducted at MRH that provides healthcare services to the King Sabata Dalindyebo (KSD) sub-district municipality in the Eastern Cape Province of South Africa. Covid-19 screening and testing in the KSD facilitated through all clinics, community healthcare centre, one district, one regional, one central hospital and two private hospitals. MRH is one of the designated referral and management care centres for patients with COVID-19 in the Eastern Cape Province. This is a 302-bed hospital that provides a range of routine and emergency healthcare services. The hospital offers level one and two healthcare services to a catchment area consisting of approximately half a million people.

King Sabata Dalindyebo sub-district municipality is rural and one of the poorest districts in South Africa. The unemployment rate is about 35% and the community is heavily dependent on social welfare grants. Most people use state health facilities for healthcare services, and only around 4.6% have medical insurance.^11^

### Data collection

This study examined the hospital records of COVID-19 inpatients. The data were extracted for all COVID-19 patients who were hospitalised at MRH during the study period. Information on age, gender, comorbidities, duration of hospitalisation and the outcome of the patient (recovered or died) was collected. An Excel spreadsheet was used to extract the data. Ten per cent of the data collected from the records were rechecked for accuracy and quality assurance.

### Data analysis

The data analysis was performed with the Statistical Package for Social Sciences (SPSS) version 18. Frequencies with proportions were reported for categorical variables and means with standard deviations were reported for continuous variables. Clinical outcomes were sub-categorised by age, gender and co-morbidity. Furthermore, a chi-squared test was used to analyse the association of factors with the outcomes.

### Ethical considerations

Ethics clearance was obtained from the Human Research and Ethics Committee of the Walter Sisulu University (Reference number: 098/2020). Permission to conduct the study was also obtained from the Eastern Cape Department of Health (Reference number: EC_202010_027) and hospital management.

## Results

Of the 1814 patients tested for COVID-19 between 20 March and 31 July 2020, 392 patients (21.6%) tested positive, and two-thirds (65.4%) were female. Eight samples were rejected as insufficient and eight test results were undetermined.

About two-thirds (*n* = 242) of COVID-19 positive patients were hospitalised. Two of these patients were transferred to the next level of care, whilst one-third (150) were quarantined at home. The mean age of patients who tested for COVID-19 was 42.6 ± 15 years and there was no difference between males and females. The mean age of hospitalised COVID-19 positive patients was 55.5 ± 14 years and the mean age of hospitalised patients who died from COVID-19 was much higher than that of recovered patients. The mortality rate amongst hospitalised patients was 46% and the mean time spent in hospital before death was 3.53 ± 4 days. About one-third (30.4%) of them died within an hour on the day of admission. Table 1 describes the mean age of patients in different categories.

**TABLE 1:** The mean age in different categories of hospitalised COVID-19 patients at Mthatha Regional Hospital.

Tested for COVID-19	Total number	Mean age (years) ± s.d.
Total tested	1814	42.6 ± 15.19
Tested negative	1406	41.6 ± 14.68
Tested positive	392	45.5 ± 16.19
Positive hospitalised and recovered	130	49.58 ± 16.79
Positive hospitalised and died	112	61.39 ± 13.17

s.d., standard deviation; COVID-19, coronavirus disease 2019.

**FIGURE 2:**
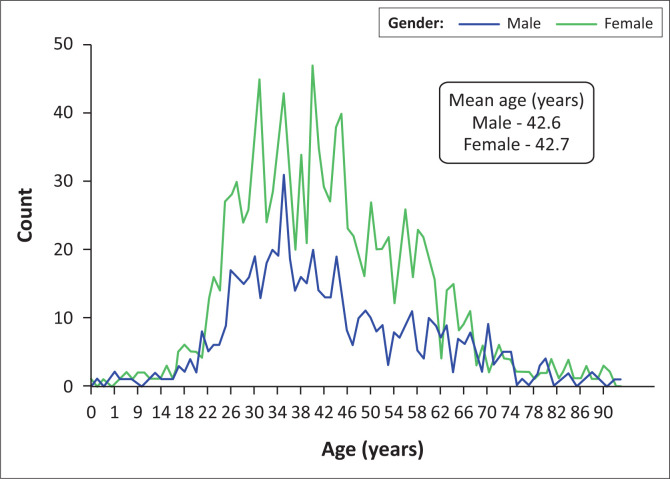
Age distribution of patients tested for COVID-19 (*n* = 1814).

Figure 2 demonstrates the age distribution amongst male and female patients.

The overall positivity rate of the COVID-19 test was 21.6%; it increased from 2.6% in April 2020 to 35% during July 2020. The mean age of patients tested for COVID-19 also increased constantly over time from March 2020 to July 2020. Table 2 demonstrates the monthly distribution of the positivity rate and the mean age of the patients tested for COVID-19.

**TABLE 2:** Monthly distribution of patients tested for COVID-19 at Mthatha Regional Hospital (*n* = 1814).

Month	Patients tested (*n*)	Mean age (years) ± s.d.	Tested positive	Positivity rate (%)
March	2	39.50 ± 17.67	1	50.0
April	112	39.17 ± 16.11	3	2.6
May	788	41.39 ± 13.58	85	10.7
June	710	44.33 ± 15.66	232	32.8
July	202	43.81 ± 18.05	71	35.1

**Total**	**1814**	**42.67 ± 15.19**	**392**	**21.6**

s.d., standard deviation.

Overall, 188 (77.6%) hospitalised patients had clinical comorbidity on admission. Diabetes (36.8%) was the most common comorbidity, followed by hypertension (33.1%), HIV (10.7%), lower respiratory tract infection (LRTI) (9%) and other comorbidity (8.6%), including cerebrovascular accident, epilepsy and Down’s syndrome.

**FIGURE 3:**
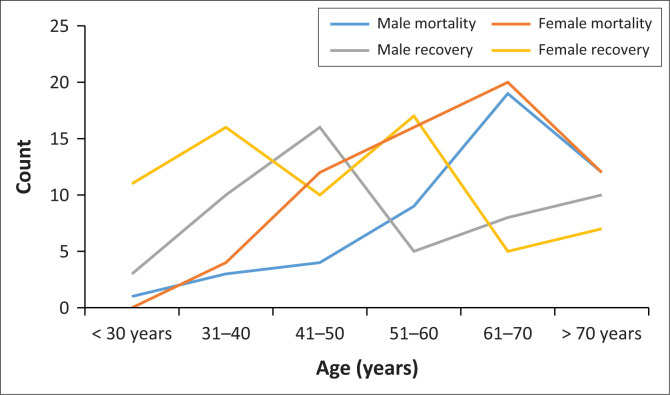
Gender and age distribution of COVID-19 outcomes (*n* = 242).

The mortality rate amongst hospitalised patients increased with age in both genders. The highest mortality was in the age group of 61–70 years. There was no difference between the two genders in the recovery rate across the different age groups. The recovery rate amongst the elderly (above 60 years) was lower, compared to younger age groups in both genders. Figure 3 compares the clinical outcomes amongst hospitalised patients based on their age and gender at MRH.

**FIGURE 4:**
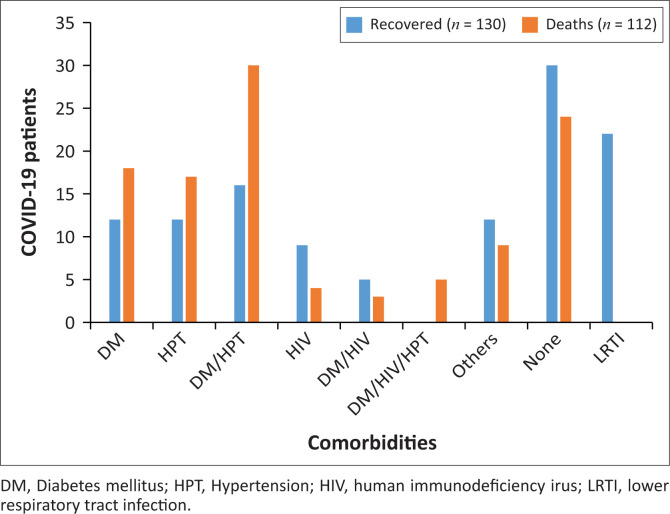
Clinical outcomes of COVID-19 hospitalised patients and associated comorbidity at Mthatha Regional Hospital (*n* = 242).

Diabetes was the most common comorbidity associated with higher mortality amongst COVID-19 hospitalised patients and the risk increased exponentially when diabetes, hypertension and HIV coexisted. There was no mortality reported amongst patients presenting with LRTI as comorbidity during hospitalisation. About half of the patients who were hospitalised without any comorbidity died because of COVID-19-related complications. Figure 4 compares the comorbid conditions amongst hospitalised patients according to their clinical outcomes.

## Discussion

This study describes the clinical outcomes of hospitalised patients with COVID-19 in a regional hospital during the peak of the epidemic in South Africa. Although the COVID-19 infection affects people across all age groups, the mean age of hospitalised patients was higher and the mean age of hospitalised patients who died was even higher. Elderly people developed severe disease and had a significant risk of mortality compared with their younger counterparts. Advanced age was an independent factor associated with the poor outcome of the hospitalised patient and there was no difference between male and female patients. These findings are in agreement with studies reported around the world.^12,13,14^

The finding of our study highlighted that most hospitalised COVID-19 patients who died were of 60 years and older. Overall, the mortality rate amongst hospitalised patients was 46%, which is much higher than the national (18.3%) and Eastern Cape provincial rate (27.5%) reported in the sentinel hospital surveillance report of the National Institute of Communicable Disease (NICD).^15^ The mortality rate was more than double amongst the elderly than amongst their younger counterparts. More than half of the patients who required hospitalisation were elderly, which was consistent with prior findings of disproportionate COVID-19 severity with advanced age.^12^ In general, our data showed that mortality increased with age and the coexistence of other medical conditions. The national surveillance system for COVID-19 hospitalisations conducted by the NICD reported age and underlying medical conditions as potential risk factors for COVID-19 in-hospital mortality.^16^ Similar findings were reported from a meta-analysis of clinical characteristics of covid-19 patients in Africa where clinical outcomes directly correlate with the underlying medical conditions and age.^17^ Furthermore, this high mortality rate amongst the elderly with comorbidity was also reported from many international studies from France, the United Kingdom and Germany in the same age groups.^12,18^

Our data also indicated that the majority of COVID-19 hospitalised patients who died during this period had underlying medical comorbidity and one-third of them died either on arrival to the hospital or within 1 h of arrival. The high rate of COVID-19 mortality was the cumulative effect of the sudden surge in the infection rate during the peak of the epidemic, coupled with high hospitalisation of COVID-19 patients, putting additional pressure on bed and oxygen availability in the hospital. The sudden surge in infection precipitated a mismatch between the availability of health resources and the need for such resources.^12,19^

Our study also noted that patients accessed health services at the hospital very late. The stigma associated with the COVID-19 outbreak, poor access and lack of knowledge of self-management may have contributed to a late presentation by patients. Several studies have reported the negative attitudinal reactions of society about many physical and psychological health problems, such as HIV/AIDS and mental illnesses, where the stigmatised become the passive recipients of negative emotional reactions from others. Although the stigma associated with the COVID-19 pandemic has not been a well-established phenomenon because of its contextual nature, the way it unfolds might vary depending on the specific context.^20,21^ The COVID-19 stigma increased the risk of social and economic isolation, discrimination and marginalisation across the community. This behaviour not only increased the risk of community transmission but also contributed to high mortality amongst COVID-19 owing to late presentation.

National and international COVID-19 research indicates a well-established direct relationship between underlying comorbidities and the clinical outcome of hospitalised patients.^12,18^ Our study reported a high baseline prevalence of clinically significant comorbidity amongst hospitalised patients. Diabetes was the most common comorbidity associated with mortality amongst hospitalised COVID-19 patients at MRH. Globally, the association between diabetes and increased morbidity and mortality from COVID-19 is reported by many studies.^19^ Our study reported a large number of hospitalised COVID-19 patients with diabetes, as well as people presenting with new onset of diabetes.

The other common comorbidities reported amongst COVID-19 hospitalised patients were hypertension and HIV. The severity of the disease was disproportionally high and it had a poor clinical outcome when more than one comorbidity coexisted.^12^ Although the study findings cannot distinguish whether the treatment or the severity of the disease accounts for the poor outcome, it supports similar findings from other studies.

## Limitation

Firstly, the source of data is limited to one public health facility only; hence, these patients do not represent all hospitalised patients with COVID-19. Therefore, the findings of this study cannot be generalised. Secondly, the patient-specific data are limited to assessment and diagnosis made by a healthcare professional. Furthermore, the study design limitations of the dataset in terms of the scope of variables on the data were collected. The findings were limited because of a lack of data availability of patients’ oxygen requirements and control of underlying comorbidity. The study findings could not differentiate whether the death was directly related to COVID-19 or an underlying medical condition.

## Conclusion

In conclusion, the majority of patients who were hospitalised for COVID-19 had high baseline comorbidities. Advance age, underlying comorbidities (diabetes, hypertension and HIV) and late presentation were associated with poor outcomes amongst hospitalised COVID-19 patients at MRH. In general, the results of the study were consistent with results published elsewhere, so Mthatha was no different, except perhaps for the phenomenon of late presentation.
